# Perceptions of oncology as a career choice among the early career doctors in Pakistan

**DOI:** 10.1186/s12909-022-03123-1

**Published:** 2022-01-26

**Authors:** Muhammad Aemaz Ur Rehman, Hareem Farooq, Muhammad Ebaad Ur Rehman, Muhammad Mohsin Ali, Amjad Zafar, Muhammad Abbas Khokhar

**Affiliations:** 1 Department of Medicine, King Edward Medical University, Neela Gumbad Chowk, Anarkali, Lahore, 54000 Pakistan; 2 Allama Iqbal Medical College, Lahore, Pakistan; 3 Department of Oncology, King Edward Medical University, Neela Gumbad Chowk, Anarkali, Lahore, 54000 Pakistan

**Keywords:** Cancer Care Facilities, Career Choice, Medical Oncology, Pakistan, Physicians

## Abstract

**Background:**

Lack of oncologists is a growing global concern. With the rise in cancer burden across the world, the supply–demand mismatch of the oncology workforce is projected to increase. Furthermore, oncology is a low-ranked field of choice among medical students, and without understanding the perceptions and concerns of early-career doctors regarding oncology, any investments made in cancer care will be futile. This study aims to determine the opinions of young doctors and the factors most affecting their preferences in order to devise focused strategies to attract more doctors into oncology.

**Methods:**

A cross-sectional study was conducted on 300 early-career doctors across various public and private hospitals in Pakistan, from March to November 2019. A close-ended, self-administered questionnaire was used to assess their opinions in terms of the workplace environment, scope, and the emotional and financial aspects of oncology. Data was analyzed using SPSS version 23 and the influence (positive or negative) of the perceptions on the choice of oncology as a career was determined by binary logistic regression analysis.

**Results:**

Almost three-quarters of the participants did not want a career in oncology. The top positive perceptions about oncology in descending order were: progressive field, gender-neutral, stable working hours, financially healthy, and work-family balance. Top negative perceptions were: lack of oncologic facilities in hospitals, radiation exposure, need for private practice, poor patient prognosis, high patient load, and depressing environment. Participants who attended private medical school (p < 0.10), planned to live abroad (p < 0.10), had an oncologist (p < 0.05), cancer survivor or death due to cancer in the family (p < 0.05), were more likely to adopt oncology as a career. Those who believed that poor patient prognosis can have an impact on career choice were less likely to prefer oncology (p < 0.05).

**Conclusion:**

Despite the rising cancer burden, early career doctors are reluctant to join oncology. Curricular, infrastructural and policy changes are needed at the level of medical school, oncology training and practice to recruit more young doctors and minimize the existing paucity of the oncologic workforce.

**Supplementary Information:**

The online version contains supplementary material available at 10.1186/s12909-022-03123-1.

## Background

Despite significant advances in the field of medicine, cancer remains both a mystery and a challenge to the healthcare systems across the world. The GLOBOCAN report identified 18.1 million new cancer cases, 9.6 million cancer deaths, and 44 million people living with cancer (within five years of diagnosis) across the world in 2018 [1]. The latest figures suggest that more than one in two people born after 1960 in the UK will develop cancer during their lifetime [2]. The United States is likely to suffer from over 608 thousand deaths due to cancer in the year 2021, corresponding to over 1600 deaths per day [3]. More cancer specialists will be needed to cater to this cancer burden as it continues to rise. Various studies in Europe, Australia, and the USA have previously highlighted the discordance between supply and demand of the oncologic workforce [4–7]. A report submitted to the American Society of Clinical Oncology (ASCO) by the AAMC warned that the nation is expected to face an acute shortage of oncologists by the year 2020, and called for a concerted approach to solve this problem. [6]

Pakistan is the fifth most populous country in the world and its cancer statistics are no different from the developed world. Pakistan had 173 thousand new cancer cases, more than 118 thousand deaths each year, and more than 310 thousand five-year prevalence as of GLOBOCAN report 2018.[1] Because of relatively poor preventive measures, lack of awareness and exposure to risk factors like household solid fuel use, physical inactivity, obesity, tobacco, poor lifestyle conditions etc., the Pakistani population is predisposed to cancer [8]. The density of physicians per 10,000 population is 7.8 [9] while the global standards require around 20 physicians per 10,000 population. The number of oncologists among these already scarce physicians is alarmingly low [10]. There are approximately 26 facilities only, including the private and public sectors, to manage such a huge number of new and existing cancer patients across the country. For a population of above 201 million [1], approximately 125 trained oncologists are available all over the country with different levels of qualification in oncology. [10].

Any investments made in cancer care without addressing the cancer workforce are likely to fail. A nationwide study conducted across the UK revealed a mean oncology exposure of only two weeks among medical students and a gross shortage of postgraduate oncology training spots in the country [11]. Moreover, many studies conducted globally and in Pakistan reveal that oncology is not a preferred field of choice among medical students [12–18]. To the best of our knowledge, all studies that have raised this issue so far have focused primarily on oncologists in training or practice and have tried to acquire their views and experiences regarding oncology as a career [7, 19]. We propose that targeting early career doctors who are yet to choose their speciality is a more prudent approach as their opinions are less likely to be biased by personal work experiences; any infrastructural change (e.g. increase in oncology training spots) that does not take into account the opinions of major stakeholders is likely to fail. Therefore, in addition to determining the perceptions of early-career doctors in general, this study will also identify the most influential factors that impact their choice against or in favour of oncology. This will help identify areas that need to be improved in order to align the trainees’ preferences and perceptions with the environment of oncology and attract a greater fraction of emerging doctors.

## Material and methods

### Study characteristics

This cross-sectional study was conducted from March to November 2019 in various public and private sector hospitals located in Lahore, Pakistan. The majority of responses were collected from Mayo Hospital and Jinnah Hospital, two of the largest hospitals in South-East Asia. These are approximately 3000 bedded hospitals that induct doctors from all over the country and abroad, and cater to a huge patient influx. Some of the private sector hospitals included Fatima Memorial and Combined Military Hospital (CMH). Non-probability convenience sampling was used.

### Study population (inclusion and exclusion criteria)

**Fig. 1:**
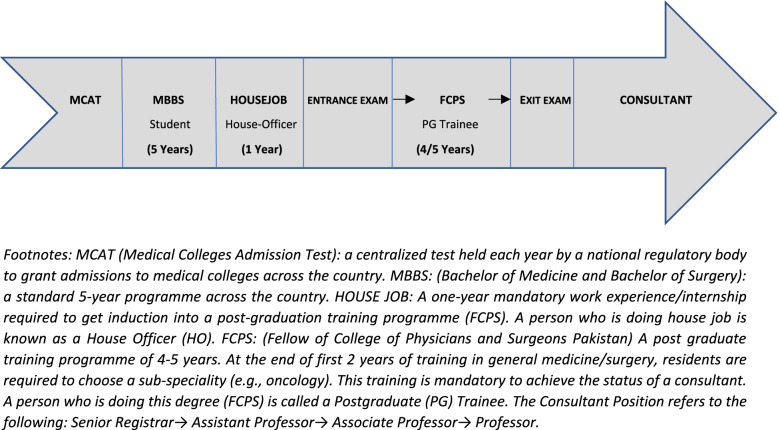
Medical Career Timeline in Pakistan

The target population for this study was early career doctors i.e. Internees/House Officers (HOs) and early Postgraduate Trainees (PGY-1 and PGY-2) who were yet to choose a speciality (Refer to Fig. 1 for details of medical career timeline in Pakistan). Postgraduate trainees who were already training in a particular speciality (PGY-3, PGY-4, PGY5) and oncologists in practice were excluded from our study because their perceptions are more likely to be biased by practical and personal experiences of working in a particular speciality. Medical students were excluded because they are at the opposite end of the career spectrum, with limited acuity and clinical exposure to adequately interpret and answer the survey items.

### Ethical considerations

This study was approved by the Institutional Review Board of King Edward Medical University (No.174/RC/KEMU; attached in related files), including procedures to protect participants' rights and privacy, and complied with the ethical principles contained in the Declaration of Helsinki (1964) and its later amendments. Data was collected from those individuals who gave informed consent to participate in the study; it was used solely for research purposes and the responses were kept confidential and anonymous.

### Sample size and cooperation rate

Participants who met the inclusion criteria set by the authors were recruited in the study. A total of 325 doctors were approached to participate in the study out of which 18 refused to give consent. Of the remaining 307 who consented to participate, seven left the questionnaire incomplete (and were thus excluded) leaving behind a total of 300 participants. Hence, the cooperation rate was 92.3%. No imputation method was used and only those 300 participants were included in the study whose complete data was acquired.

### Questionnaire

All the participants were asked to fill a self-administered, close-ended, easy-to-interpret questionnaire. The authors ran a preliminary literature search, critically appraised the previously published studies, and after an elaborate discussion, a detailed questionnaire was designed for this study. The questionnaire was developed in the English language as the target population was doctors whose medium of study was English. Before being administered, it was studied in-depth by two oncologists and public health specialists working in Mayo Hospital and King Edward Medical University, respectively who declared the questionnaire as valid. It was further studied by a native English speaker who confirmed it for linguistic validity. The questionnaire consisted of three sections: The first section inquired about the demographic variables such as age, gender, medical school, working hospital (public vs private), and current training position. The second part of the questionnaire contained questions to gauge the participants’ perception of oncology in terms of the workplace environment, the finances, the emotional aspect, the scope of the field, and the nature of the work. All of these responses were recorded through a three-point scale (agree, disagree, neutral/rather not say). The third section had questions regarding the participants’ prior working experience and future interest in oncology.

### Data collection and bias reduction

Two authors (MAUR and HF) administered the questionnaire to all participants for data collection. The interaction with the respondents began with a formal introduction and then proceeded towards the explanation of the questionnaire. Consent was taken from all participants, who were informed that the responses were anonymous and that they were free to withdraw at any time. The research lead (MAK) trained MAUR and HF on how to approach participants in order to eliminate any potential bias. Participants were left alone to fill the questionnaire in order to minimize any external influence or coercion.Statistical analysis.

Data was analyzed using IBM SPSS Statistics software for Windows Version 23. Continuous variables like age are presented as mean ± S.D. Categorical variables like demographics and perceptions are presented as frequencies and percentages. The influence of these perceptions on the choice of oncology as a career is determined by binary logistic regression analysis, the results of which are presented as Odds Ratio (OR) with a 95% Confidence Interval.

## Results

A total of 300 doctors in their early career years completely answered all the survey items. The number of male (*n* = 138, 46%) and female respondents (*n* = 162, 54%) were almost comparable, with more than three quarters belonging to public sector (government) medical schools. The mean age of the participants was 25.45 ± 2.27 years. Almost ¾ participants said that they will not choose oncology as a career if given a chance to decide (Table 1).

**Table 1:** Demographic features of study participants

Variable	*N* (%)
**Gender**	
Male	138 (46.0%)
Female	162 (54.0%)
**Stage of Medical Career**	
House Officers/Internees	204 (68.0%)
Post Graduate Trainees	96 (32.0%)
**Medical School**	
Government/Public Sector	252 (84.0%)
Private/Foreign Sector	48 (16.0%)
**Will prefer oncology in future if given a chance to decide**	
Yes	82 (27.3%)
No	218 (72.7%)
**History of cancer diagnosis or death due to cancer in family**	
Yes	153 (51.0%)
No	111 (37.0%)
Rather not say	36 (12.0%)
**Presence of oncologist in family**	
Yes	26 (8.7%)
No	274 (91.3%)
Rather not say	0 (0%)

The top three negative perceptions (Table 2) of oncology were found to be a lack of oncologic facilities in hospitals (70%), the fear of radiation exposure (66%), and the need for private practice to meet financial needs (61%). The poor prognosis of cancer patients (57%), high patient load (56%) and depressing nature of the field (54%) were some of the other notable concerns. The most popular positive perceptions about oncology in the descending order were: the progressive nature of the field (85%), gender-neutral speciality (77%), stable working hours (61%), financially rewarding (48%), and the presence of work-family balance (47%). The perceptions of male and female doctors were almost aligned with slightly more (non-significant) females acknowledging the depressing nature of oncology and concern for radiation exposure (Table 2).

**Table 2:** Perceptions of Oncology Among Early Career Doctors in Pakistan

Perception of Oncology as a Career Choice Among Early Career Doctors in Pakistan			
	**Agree**	**Disagree**	**Neutral**
**Workplace Environment**			
Oncology is a male oriented specialty	31 (10.3%)	231 (77.0%)	38(12.7%)
There is work-family balance	140 (46.7%)	53(17.7%)	107 (35.7%)
Working hours are stable	183 (61.0%)	29 (9.7%)	88 (29.3%)
High patient load in oncology	167 (55.7%)	50 (16.7%)	83 (27.7%)
There is lack of proper oncologic facilities in Pakistani hospitals	210 (70.0%)	52 (17.3%)	38(12.7%)
**Financial Aspect**			
A financially healthy specialty	143 (47.7%)	62 (20.7%)	95 (31.7%)
Will need private practice to suffice financial requirements	182 (60.7%)	49 (16.3%)	69 (23.0%)
**Emotional Aspect**			
Oncology is a depressing field	163 (54.3%)	61 (20.3%)	76 (25.3%)
Long term patient affiliation is energy consuming	110 (36.7%)	117 (39.0%)	73 (24.3%)
**Scope of the Field**			
Saturation/Less job opportunities in oncology	80 (26.7%)	147 (49.0%)	73 (24.3%)
A progressive and research-oriented field	256 (85.3%)	17 (5.7%)	27 (9.0%)
**Nature of the Work**			
Radiation exposure is a concern while working in oncology	198 (66.0%)	48 (16.0%)	54 (18.0%)
Poor patient prognosis impacts career choice	170 (56.7%)	72 (24.0%)	58 (19.3%)

OR- Odds Ratio

CI- 95% Confidence Interval

**Fig. 2:**
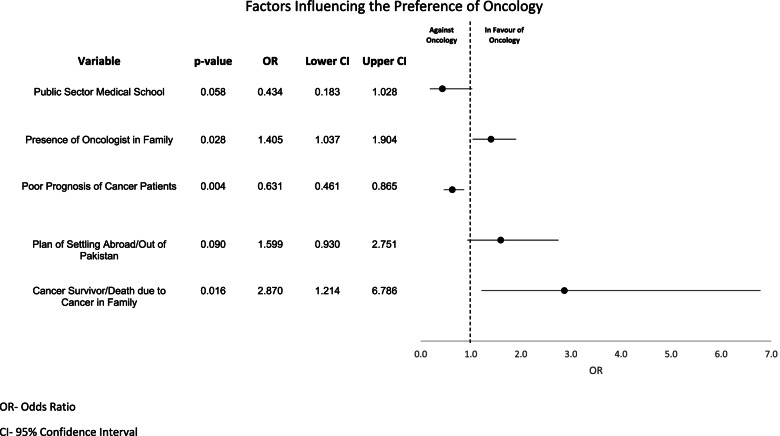
Binary Logistic Regression of Factors Influencing the Preference of Oncology as a Career

Binary logistic regression (Fig. 2) assessed the association between these perceptions and the preference of oncology as a career. The participants who believed that poor patient prognosis can have an impact on their career choice were more likely to not prefer oncology as a career (p < 0.05). There was a lesser tendency amongst graduates of government medical schools (vs private medical schools) to opt for this field (p < 0.10), along with those who plan to settle in Pakistan (vs abroad) in future (p < 0.10), although these findings did not reach statistical significance. On the other hand, the presence of an oncologist(s) in the family (p < 0.05) and having had a cancer survivor or death due to cancer in the family (p < 0.05) was associated with a significantly higher likelihood of choosing oncology as a potential career.

## Discussion

There is a significant paucity of global literature regarding the perceptions of doctors about oncology as a field [7, 19]. Their decision about choosing oncology as a career in the future is likely to be based on or influenced by their perceptions i.e. what they think of oncology as a field. Many studies across the world have not found oncology as a preferred field of choice among medical students [12–18]. The reluctance to opt for oncology is alarming because of the rising cancer burden and the mismatch between supply and demand (of oncologists) that results from it. This mismatch has hampered the quality of patient care, as specialised care provided by oncologists is sparse, and hence primary care physicians have to provide a major share of cancer care [11, 20]. Recognising this problem, we surveyed the early career doctors in Pakistan to determine their raw perceptions and identified the likely contributing factors in the choice against or for oncology. As expected, the results showed an aversion to oncology in most (¾) early career doctors. The poor patient prognosis (and the likely emotional fix associated with it) was one of the primary factors contributing to this disinclination. On the other hand, the presence of an oncologist in the family, cancer survivor or death due to cancer in the family, and future plan to live abroad (out of Pakistan) was associated with a preference for oncology in the future.

Oncology is a highly competitive and progressive field. The research-oriented nature of the field, the ever-growing literature, and the periodic emergence of newer chemotherapeutic drugs require oncologists to keep up with the medical literature [21–26]. The drive towards distinction, largely gained through substantive research credentials or higher qualifications, the dearth of good jobs and opportunities, and the need to ‘keep up’ are some of the tough experiences described by trained oncologists [26]. Although Pakistan has scarce research trends as compared to the developed countries, yet the competitive nature of the field, the shortage of opportunities, peer pressure, and the need to excel are nearly universal. The majority of the respondents in our study believed that oncology is a highly progressive and challenging speciality (Table 2) but whether or not this impacted their choice was not clear. If considered from a positive viewpoint, this perception can mean that young doctors either believe that Pakistan lacks the modern facilities and research culture to cope up with the progression this field demands or that their other negative perceptions outweigh any positive impact this perception could have had on their career choice. On the other hand, another interpretation that could be derived from this perception is that the inherently challenging nature of oncology acts as a deterrent rather than an impetus.

The paucity of the oncologic workforce is a global problem, extending to developed countries like the USA which expects a shortage of 2,550 to 4,080 oncologists between the years 2005 and 2020 [5]. Medical Oncology Group of Australia (MOGA) has reported and predicted shortfall in the past [4], the National Chemotherapy Advisory Group (NCAG) in the UK has reported that the cancer care needs of the community are not being met [11] and France predicts a dramatic decrease in oncologists during the next five years [7]. The biggest factors contributing to the discrepancy between supply and demand of oncologic services are the shortage of oncology training slots, the disinclination of medical students and early-career doctors to oncology, and the lack of dedicated cancer facilities [5]. Circumstances in Pakistan are no different; it faces a similar supply–demand mismatch. According to the latest statistics, there are only 20 dedicated cancer facilities and 50 other hospitals which care for cancer patients [10]. There are only 125 trained oncologists, 25 radiotherapy machines, 6 oncology-centric conferences all over Pakistan and Pakistan is yet to publish a national oncology journal [10]. There is a gross deficit of oncological services in its most populous province ‘Punjab’, with the ratio of medical oncologists to the population being 0.027 per 100,000 [27]. The increasingly competitive nature of oncology as discussed previously, along with lack of substantial research opportunities and inadequate learning avenues (e.g. oncology conferences) in Pakistan [28] hamper a physician’s growth and result in an inability to effectively compete with international peers. All these factors when combined with the lack of slots available in Pakistan might explain the association found between the preference of oncology as a career and the decision to live or work abroad.

Since the beginning of time, no disease has brought more affliction to the human race than cancer. Cancer is a family experience; when it embraces an individual, it embraces a family [29]. The family members follow the phases of the disease, very often suffering comparable or greater distress than the patient. It transforms the personalities and thought processes of the patient as well as his/her loved ones [29, 30]. Grief is one of the most powerful human emotions; seeing your loved ones go through it can have a strong impact on the human psyche. Our study found that those who had had a cancer survivor or death due to cancer in their family were more likely to choose oncology as a career in the future. As much as a loss or suffering leaves you dejected, it may also instil in you the wish to heal the suffering of fellow cancer-stricken or the psychological will to overcome what has defeated or tormented you in the past. Emotions have been shown to play a significant role in choosing a career in oncology and a qualitative study done on Australian oncologists in 2016 stated that ‘the tussle between *importance of intimacy* and *art of detachment* is one of the core qualities of this field and the uniqueness that draws people into it’ [7, 26]. It is important to mention here that the family system in Pakistan is much more strong as compared to the Western world; most people live in joint families and share the same home, therefore the possibility that this factor may have played some role in the association found here can not be ignored. Interestingly, our study also found that having an oncologist in the family was associated with a greater likelihood of pursuing oncology, a finding that further stresses the influential role of families in the East.

The monetary compensation provided by any field is a factor that cannot be ignored when it comes to opting for a career [31–33]. A study conducted by Khan AH identified low pays and lack of promotions as primary contributors to high employee turnover rate and lack of job satisfaction [34]. Medscape report published in 2020 ranked oncology amongst the top 10 highly paid specialities, and most of the early career doctors in our study also believed that oncology is financially rewarding [35]. Despite this notion, it was concerning to note that a high percentage of doctors also believed that they would need private practice in order to satisfy their financial needs. A potential reason for this could be low salaries and job insecurity in the government sector of Pakistan [36, 37] therefore the need to work in the private sector is a common practice across all fields. All these findings highlight the need for policy change and infrastructure development in oncology, especially in the public sector.

### Strengths

The strength of our paper lies in the fact that it was conducted solely on early career doctors who have graduated but are yet to choose their field for specialization. Despite the global nature of this issue, only a handful of studies have been published on this topic so far, and all of them have presented oncology through the lens of oncologists. To the best of our knowledge, this study is the first of its kind, in the world and Pakistan, to gauge the mindset of young doctors regarding oncology. Our study included the biggest and most populous hospitals having doctors from all across the country, hence ensuring adequate representation. We evaluated numerous characteristics of oncology in greater depth, as compared to other studies conducted on the topic across the world, ranging from the workplace environment and scope of the field to the nature of the work and emotional aspects.

### Limitations

The authors would like to acknowledge some limitations in the work that is presented. Although the study population was representative, the sample size was still small relative to the number of doctors in the country. Non-probability convenience sampling technique was used to collect the data, which is a non-randomized sampling method that has the potential for selection bias. No standardized questionnaire was available on the subject, so a tailored questionnaire had to be designed by a team of specialists after reviewing the literature. Lastly, the survey questions were close-ended with a special focus on factors that can play a role in the choice of oncology as a career; a mixed-methods approach would have aided in acquiring a wider range of opinions and recommendations from early career doctors.

### Implications and recommendations

Our results indicate a clear disinclination among early-career doctors towards oncology as a career. A concerted approach that involves changes at the level of basic medical education, postgraduate training, and specialised oncology practice, is needed to solve this problem. The practical issues described by many doctors regarding lack of oncologic facilities and radiation exposure take priority as these concerns qualify as basic workplace needs. Increasing the post-graduate training spots in oncology is the need of the hour- a step that will eventually bridge the supply–demand mismatch [11]. This will not only provide employment security to future oncologists but will also help create work-life balance by properly distributing the ever-growing patient load. Moreover, keeping in view the human tendencies, the role of greater monetary incentives to drive more doctors to oncology cannot be ignored.

A lot of repulsion to oncology comes from the poor prognosis of cancer patients, which creates an emotional predicament for doctors in addition to the patient and family. Emotional challenges and the depressing nature of the field can be countered by focused training workshops at a postgraduate level (e.g. residency and fellowship) that teach oncology-specific patient interaction (e.g. how to break the news of cancer to patients and families) and communication during palliative care. The inclusion of such workshops within the curricula is the most effective way to hone these skills. The ‘depth and variety of human relations’ has been labelled as one of the most important factors in choosing oncology by many oncologists [26], therefore, if used wisely, the emotional aspect of this field can be used to attract (rather than repel) more doctors.

At the level of medical school, the primary problem is the lack of undergraduate exposure to oncology, with only a limited part of the curriculum dedicated to oncology and inadequate oncology clerkship requirements [2, 11, 38]. Well-designed oncology conferences and didactic clinical placements (rotations) have been shown to boost interest in oncology [39]. Medical school oncology interest groups (OIGs) have also been found to promote interest in the field and improve confidence in breaking bad news [40]. Currently there is non-uniformity in curricula and variability in the duration of undergraduate oncology exposure [11], and a comprehensive review is needed to identify deficiencies in the structure of basic medical education.

## Conclusion

There is a significant supply–demand mismatch of the oncologic workforce globally but the young doctors are still reluctant to join oncology. Doctors who plan to live abroad (out of Pakistan) in the future, have an oncologist in their family, and have had a cancer survivor or death due to cancer in the family are more likely to choose oncology as a career. However, those who believe that poor patient prognosis can have a significant impact on their career choice are less likely to prefer oncology. Identifying the reasons behind the lack of preference and doing a comprehensive analysis in order to make efforts in the right direction is the first step in dealing with the cancer burden worldwide and ensuring the attractiveness of physicians to this long-deserted field. Policy changes in medical education, as well as oncology practice, are needed to solve this problem.

## Supplementary Information

**Additional file 1.** Binary Logistic Regression of Factors Influencing the Preference of Oncology as a Career.

**Additional file 2.** Frequencies and Percentages.

**Additional file 3.** Demographics.

## Data Availability

All data generated or analyzed during this study are included in the supplementary files.
