# Supplementary material for: Perceptions of oncology as a career choice among the early career doctors in Pakistan
Source: BMC Med Educ. 2022 Jan 26;22:61. doi: 10.1186/s12909-022-03123-1 (PMC8790828; doi:10.1186/s12909-022-03123-1)
Supplement: Supplementary file 1 — Additional file 1. Binary Logistic Regression of Factors Influencing the Preference of Oncology as a Career. [file 12909_2022_3123_MOESM1_ESM.docx]

**Supplementary File 1: Binary Logistic Regression of Factors Influencing the Preference of Oncology as a Career**

| **Case Processing Summary** | | | |
| --- | --- | --- | --- |
| Unweighted Cases^a^ | | N | Percent |
| Selected Cases | Included in Analysis | 300 | 100.0 |
|  | Missing Cases | 0 | .0 |
|  | Total | 300 | 100.0 |
| Unselected Cases | | 0 | .0 |
| Total | | 300 | 100.0 |

| a. If weight is in effect, see classification table for the total number of cases. |
| --- |

| **Dependent Variable Encoding** | |
| --- | --- |
| Original Value | Internal Value |
| No | 0 |
| Yes | 1 |

**Block 0: Beginning Block**

| **Classification Table^a,b^** | | | | | |
| --- | --- | --- | --- | --- | --- |
|  | Observed | | Predicted | | |
|  |  |  | Will you prefer oncology as your career if you are given a chance to decide? | | Percentage Correct |
|  |  |  | No | Yes |  |
| Step 0 | Will you prefer oncology as your career if you are given a chance to decide? | No | 218 | 0 | 100.0 |
|  |  | Yes | 82 | 0 | .0 |
|  | Overall Percentage | |  |  | 72.7 |

| a. Constant is included in the model. |
| --- |
| b. The cut value is .500 |

| **Variables in the Equation** | | | | | | | |
| --- | --- | --- | --- | --- | --- | --- | --- |
|  | | B | S.E. | Wald | df | Sig. | Exp(B) |
| Step 0 | Constant | -.978 | .130 | 56.968 | 1 | .000 | .376 |

| **Variables not in the Equation** | | | | | |
| --- | --- | --- | --- | --- | --- |
|  | | | Score | df | Sig. |
| Step 0 | Variables | qHrelative | 10.075 | 1 | .002 |
|  |  | qGabroad | 4.327 | 1 | .038 |
|  |  | qE2prognosis | 8.462 | 1 | .004 |
|  |  | qC1family | 4.698 | 1 | .030 |
|  |  | q5university | 3.273 | 1 | .070 |
|  |  | q6training | 5.552 | 1 | .018 |
|  | Overall Statistics | | 31.727 | 6 | .000 |

**Block 1: Method = Enter**

| **Omnibus Tests of Model Coefficients** | | | | |
| --- | --- | --- | --- | --- |
|  | | Chi-square | df | Sig. |
| Step 1 | Step | 32.750 | 6 | .000 |
|  | Block | 32.750 | 6 | .000 |
|  | Model | 32.750 | 6 | .000 |

| **Model Summary** | | | |
| --- | --- | --- | --- |
| Step | -2 Log likelihood | Cox & Snell R Square | Nagelkerke R Square |
| 1 | 319.178^a^ | .103 | .150 |

| a. Estimation terminated at iteration number 5 because parameter estimates changed by less than .001. |
| --- |

| **Classification Table^a^** | | | | | |
| --- | --- | --- | --- | --- | --- |
|  | Observed | | Predicted | | |
|  |  |  | Will you prefer oncology as your career if you are given a chance to decide? | | Percentage Correct |
|  |  |  | No | Yes |  |
| Step 1 | Will you prefer oncology as your career if you are given a chance to decide? | No | 207 | 11 | 95.0 |
|  |  | Yes | 68 | 14 | 17.1 |
|  | Overall Percentage | |  |  | 73.7 |

| a. The cut value is .500 |
| --- |

| **Variables in the Equation** | | | | | | | |
| --- | --- | --- | --- | --- | --- | --- | --- |
|  | | B | S.E. | Wald | df | Sig. | Exp(B) |
| Step 1^a^ | qHrelative | 1.054 | .439 | 5.764 | 1 | .016 | 2.870 |
|  | qGabroad | .469 | .277 | 2.876 | 1 | .090 | 1.599 |
|  | qE2prognosis | -.460 | .160 | 8.220 | 1 | .004 | .631 |
|  | qC1family | .340 | .155 | 4.813 | 1 | .028 | 1.405 |
|  | q5university | -.834 | .440 | 3.599 | 1 | .058 | .434 |
|  | q6training | -.328 | .188 | 3.035 | 1 | .082 | .720 |
|  | Constant | -.078 | .851 | .008 | 1 | .927 | .925 |

| a. Variable(s) entered on step 1: qHrelative, qGabroad, qE2prognosis, qC1family, q5university, q6training. |
| --- |
