# Supplementary material for: Perceptions of oncology as a career choice among the early career doctors in Pakistan
Source: BMC Med Educ. 2022 Jan 26;22:61. doi: 10.1186/s12909-022-03123-1 (PMC8790828; doi:10.1186/s12909-022-03123-1)
Supplement: Supplementary file 2 — Additional file 2. Frequencies and Percentages. [file 12909_2022_3123_MOESM2_ESM.docx]

***Supplementary File 2: Frequencies and Percentages***

| **Do you believe that oncology is a male-oriented speciality?** | | | | | |
| --- | --- | --- | --- | --- | --- |
|  | | Frequency | Percent | Valid Percent | Cumulative Percent |
| Valid | Disagree | 231 | 77.0 | 77.0 | 77.0 |
|  | neutral | 38 | 12.7 | 12.7 | 89.7 |
|  | Agree | 31 | 10.3 | 10.3 | 100.0 |
|  | Total | 300 | 100.0 | 100.0 |  |

| **Is there work-family life balance in oncology?** | | | | | |
| --- | --- | --- | --- | --- | --- |
|  | | Frequency | Percent | Valid Percent | Cumulative Percent |
| Valid | Disagree | 53 | 17.7 | 17.7 | 17.7 |
|  | neutral | 107 | 35.7 | 35.7 | 53.3 |
|  | Agree | 140 | 46.7 | 46.7 | 100.0 |
|  | Total | 300 | 100.0 | 100.0 |  |

| **Do you think that working hours are stable in oncology?** | | | | | |
| --- | --- | --- | --- | --- | --- |
|  | | Frequency | Percent | Valid Percent | Cumulative Percent |
| Valid | Disagree | 29 | 9.7 | 9.7 | 9.7 |
|  | neutral | 88 | 29.3 | 29.3 | 39.0 |
|  | Agree | 183 | 61.0 | 61.0 | 100.0 |
|  | Total | 300 | 100.0 | 100.0 |  |

| **Is there high patient load in oncology?** | | | | | |
| --- | --- | --- | --- | --- | --- |
|  | | Frequency | Percent | Valid Percent | Cumulative Percent |
| Valid | Disagree | 50 | 16.7 | 16.7 | 16.7 |
|  | Neutral | 83 | 27.7 | 27.7 | 44.3 |
|  | Agree | 167 | 55.7 | 55.7 | 100.0 |
|  | Total | 300 | 100.0 | 100.0 |  |

| **Does the lack of proper facilities in oncology in Pakistani hospitals influence your career choice of oncology** | | | | | |
| --- | --- | --- | --- | --- | --- |
|  | | Frequency | Percent | Valid Percent | Cumulative Percent |
| Valid | Disagree | 52 | 17.3 | 17.3 | 17.3 |
|  | neutral | 38 | 12.7 | 12.7 | 30.0 |
|  | Agree | 210 | 70.0 | 70.0 | 100.0 |
|  | Total | 300 | 100.0 | 100.0 |  |

| **Do you believe that oncology is a financially healthy speciality?** | | | | | |
| --- | --- | --- | --- | --- | --- |
|  | | Frequency | Percent | Valid Percent | Cumulative Percent |
| Valid | Disagree | 62 | 20.7 | 20.7 | 20.7 |
|  | neutral | 95 | 31.7 | 31.7 | 52.3 |
|  | Agree | 143 | 47.7 | 47.7 | 100.0 |
|  | Total | 300 | 100.0 | 100.0 |  |

| **Do you think you will need private practice to suffice your financial requirements if you choose oncology?** | | | | | |
| --- | --- | --- | --- | --- | --- |
|  | | Frequency | Percent | Valid Percent | Cumulative Percent |
| Valid | Disagree | 49 | 16.3 | 16.3 | 16.3 |
|  | neutral | 69 | 23.0 | 23.0 | 39.3 |
|  | Agree | 182 | 60.7 | 60.7 | 100.0 |
|  | Total | 300 | 100.0 | 100.0 |  |

| **Do you think there is depression for doctors in oncology?** | | | | | |
| --- | --- | --- | --- | --- | --- |
|  | | Frequency | Percent | Valid Percent | Cumulative Percent |
| Valid | Disagree | 61 | 20.3 | 20.3 | 20.3 |
|  | neutral | 76 | 25.3 | 25.3 | 45.7 |
|  | Agree | 163 | 54.3 | 54.3 | 100.0 |
|  | Total | 300 | 100.0 | 100.0 |  |

| **Do you think long term affiliation with patients is energy consuming and non-favorable for you?** | | | | | |
| --- | --- | --- | --- | --- | --- |
|  | | Frequency | Percent | Valid Percent | Cumulative Percent |
| Valid | Disagree | 117 | 39.0 | 39.0 | 39.0 |
|  | neutral | 73 | 24.3 | 24.3 | 63.3 |
|  | Agree | 110 | 36.7 | 36.7 | 100.0 |
|  | Total | 300 | 100.0 | 100.0 |  |

| **Do you think there is saturation (less job opportunities) in the field oncology?** | | | | | |
| --- | --- | --- | --- | --- | --- |
|  | | Frequency | Percent | Valid Percent | Cumulative Percent |
| Valid | Disagree | 147 | 49.0 | 49.0 | 49.0 |
|  | neutral | 73 | 24.3 | 24.3 | 73.3 |
|  | Agree | 80 | 26.7 | 26.7 | 100.0 |
|  | Total | 300 | 100.0 | 100.0 |  |

| **Do you think that oncology is a progressive, and research oriented specialty?** | | | | | |
| --- | --- | --- | --- | --- | --- |
|  | | Frequency | Percent | Valid Percent | Cumulative Percent |
| Valid | Disagree | 17 | 5.7 | 5.7 | 5.7 |
|  | neutral | 27 | 9.0 | 9.0 | 14.7 |
|  | Agree | 256 | 85.3 | 85.3 | 100.0 |
|  | Total | 300 | 100.0 | 100.0 |  |

| **Do you fear that radiation exposure can be a damage to your own health in oncology?** | | | | | |
| --- | --- | --- | --- | --- | --- |
|  | | Frequency | Percent | Valid Percent | Cumulative Percent |
| Valid | Disagree | 48 | 16.0 | 16.0 | 16.0 |
|  | neutral | 54 | 18.0 | 18.0 | 34.0 |
|  | Agree | 198 | 66.0 | 66.0 | 100.0 |
|  | Total | 300 | 100.0 | 100.0 |  |

| **Do you think poor prognosis or late presentation of patients will affect your choice of opting oncology?** | | | | | |
| --- | --- | --- | --- | --- | --- |
|  | | Frequency | Percent | Valid Percent | Cumulative Percent |
| Valid | Disagree | 72 | 24.0 | 24.0 | 24.0 |
|  | neutral | 58 | 19.3 | 19.3 | 43.3 |
|  | Agree | 170 | 56.7 | 56.7 | 100.0 |
|  | Total | 300 | 100.0 | 100.0 |  |
