# Supplementary material for: Perceptions of oncology as a career choice among the early career doctors in Pakistan
Source: BMC Med Educ. 2022 Jan 26;22:61. doi: 10.1186/s12909-022-03123-1 (PMC8790828; doi:10.1186/s12909-022-03123-1)
Supplement: Supplementary file 3 — Additional file 3. Demographics. [file 12909_2022_3123_MOESM3_ESM.docx]

***Supplementary File 3: Demographics***

| **Which is your current working hospital?** | | | | | |
| --- | --- | --- | --- | --- | --- |
|  | | Frequency | Percent | Valid Percent | Cumulative Percent |
| Valid | govt | 289 | 96.3 | 96.3 | 96.3 |
|  | private | 11 | 3.7 | 3.7 | 100.0 |
|  | Total | 300 | 100.0 | 100.0 |  |

| **What is your medical school?** | | | | | |
| --- | --- | --- | --- | --- | --- |
|  | | Frequency | Percent | Valid Percent | Cumulative Percent |
| Valid | govt | 252 | 84.0 | 84.0 | 84.0 |
|  | Private/foreign | 48 | 16.0 | 16.0 | 100.0 |
|  | Total | 300 | 100.0 | 100.0 |  |

| **What is your current training program?** | | | | | |
| --- | --- | --- | --- | --- | --- |
|  | | Frequency | Percent | Valid Percent | Cumulative Percent |
| Valid | house officers | 204 | 68.0 | 68.0 | 68.0 |
|  | pgr | 96 | 32.0 | 32.0 | 100.0 |
|  | Total | 300 | 100.0 | 100.0 |  |
| Total | | 300 | 100.0 |  |  |

| **gender of doctor** | | | | | |
| --- | --- | --- | --- | --- | --- |
|  | | Frequency | Percent | Valid Percent | Cumulative Percent |
| Valid | male | 138 | 46.0 | 46.0 | 46.0 |
|  | female | 162 | 54.0 | 54.0 | 100.0 |
|  | Total | 300 | 100.0 | 100.0 |  |

**Frequencies**

| **Statistics** | | |
| --- | --- | --- |
| Will you prefer oncology as your career if you are given a chance to decide? | | |
| N | Valid | 300 |
|  | Missing | 0 |

| **Will you prefer oncology as your career if you are given a chance to decide?** | | | | | |
| --- | --- | --- | --- | --- | --- |
|  | | Frequency | Percent | Valid Percent | Cumulative Percent |
| Valid | No | 218 | 72.7 | 72.7 | 72.7 |
|  | Yes | 82 | 27.3 | 27.3 | 100.0 |
|  | Total | 300 | 100.0 | 100.0 |  |

| **Has there been a cancer survivor or death due to cancer in your family/friends?** | | | | | |
| --- | --- | --- | --- | --- | --- |
|  | | Frequency | Percent | Valid Percent | Cumulative Percent |
| Valid | Disagree | 111 | 37.0 | 37.0 | 37.0 |
|  | Rather not say | 36 | 12.0 | 12.0 | 49.0 |
|  | Agree | 153 | 51.0 | 51.0 | 100.0 |
|  | Total | 300 | 100.0 | 100.0 |  |

| **Is there an oncologist in your family?** | | | | | |
| --- | --- | --- | --- | --- | --- |
|  | | Frequency | Percent | Valid Percent | Cumulative Percent |
| Valid | No | 274 | 91.3 | 91.3 | 91.3 |
|  | Yes | 26 | 8.7 | 8.7 | 100.0 |
|  | Total | 300 | 100.0 | 100.0 |  |
